# Explainable machine learning model for predicting the risk of significant liver fibrosis in patients with diabetic retinopathy

**DOI:** 10.1186/s12911-024-02749-z

**Published:** 2024-11-11

**Authors:** Gangfeng Zhu, Na Yang, Qiang Yi, Rui Xu, Liangjian Zheng, Yunlong Zhu, Junyan Li, Jie Che, Cixiang Chen, Zenghong Lu, Li Huang, Yi Xiang, Tianlei Zheng

**Affiliations:** 1 The First Clinical Medical College, Gannan Medical University, Ganzhou, 341000 Jiangxi Province China; 2 The Engineering Research Center of Intelligent Theranostics Technology and Instruments, Ministry of Education, School of Biomedical Engineering and Informatics, Nanjing Medical University, Nanjing, 211166 China; 3 Artificial Intelligence Unit, Department of Medical Equipment Management, Affiliated Hospital of Xuzhou Medical University, Xuzhou, 221004 Jiangsu Province China; 4 Department of Rehabilitation Medicine, Affiliated Jinhua Hospital, Zhejiang University School of Medicine, Jinhua, 321000 Zhejiang Province China; 5 Department of Oncology, The First Affiliated Hospital, Gannan Medical University, Ganzhou, 341000 Jiangxi Province China; 6 Center of Portal Hypertension, Department of Radiology, Zhongda Hospital, Southeast University, Nanjing, 210009 China; 7 Jiangsu Key Laboratory of New Drug Research and Clinical Pharmacy, College of Pharmacy, Xuzhou Medical University, Xuzhou, China; 8 School of Information and Control Engineering, China University of Mining and Technology, Xuzhou, 211166 China

**Keywords:** Machine learning, Significant liver fibrosis, Diabetic retinopathy, National Health and Nutrition Examination Survey, SHapley Additive exPlanations

## Abstract

**Background:**

Diabetic retinopathy (DR), a prevalent complication in patients with type 2 diabetes, has attracted increasing attention. Recent studies have explored a plausible association between retinopathy and significant liver fibrosis. The aim of this investigation was to develop a sophisticated machine learning (ML) model, leveraging comprehensive clinical datasets, to forecast the likelihood of significant liver fibrosis in patients with retinopathy and to interpret the ML model by applying the SHapley Additive exPlanations (SHAP) method.

**Methods:**

This inquiry was based on data from the National Health and Nutrition Examination Survey 2005–2008 cohort. Utilizing the Fibrosis-4 index (FIB-4), liver fibrosis was stratified across a spectrum of grades (F0-F4). The severity of retinopathy was determined using retinal imaging and segmented into four discrete gradations. A ten-fold cross-validation approach was used to gauge the propensity towards liver fibrosis. Eight ML methodologies were used: Extreme Gradient Boosting, Random Forest, multilayer perceptron, Support Vector Machines, Logistic Regression (LR), Plain Bayes, Decision Tree, and k-nearest neighbors. The efficacy of these models was gauged using metrics, such as the area under the curve (AUC). The SHAP method was deployed to unravel the intricacies of feature importance and explicate the inner workings of the ML model.

**Results:**

The analysis included 5,364 participants, of whom 2,116 (39.45%) exhibited notable liver fibrosis. Following random allocation, 3,754 individuals were assigned to the training set and 1,610 were allocated to the validation cohort. Nine variables were curated for integration into the ML model. Among the eight ML models scrutinized, the LR model attained zenith in both AUC (0.867, 95% CI: 0.855–0.878) and F1 score (0.749, 95% CI: 0.732–0.767). In internal validation, this model sustained its superiority, with an AUC of 0.850 and an F1 score of 0.736, surpassing all other ML models. The SHAP methodology unveils the foremost factors through importance ranking.

**Conclusion:**

Sophisticated ML models were crafted using clinical data to discern the propensity for significant liver fibrosis in patients with retinopathy and to intervene early.

**Practice implications:**

Improved early detection of liver fibrosis risk in retinopathy patients enhances clinical intervention outcomes.

**Supplementary Information:**

The online version contains supplementary material available at 10.1186/s12911-024-02749-z.

## Introduction

Cirrhosis currently ranks as the 11th most prevalent cause of mortality worldwide, claiming an estimated one million lives annually owing to its complications, thereby imposing a substantial economic burden on numerous nations [1–3]. Significant liver fibrosis is commonly recognized as a precursor to cirrhosis, marking the initial pathological progression following liver injury [4, 5]. Although liver damage may be reversible during the early phases of fibrosis [6], persistent injury exacerbates the accumulation of fibrous tissue, ultimately culminating in cirrhosis [7–9]. Evidence suggests that significant liver fibrosis is correlated with liver-related morbidity and serves as a pivotal prognostic indicator in individuals with liver ailments [10, 11]. Thus, the early identification of liver fibrosis in patients and efficacious intervention prior to progression towards significant fibrosis/cirrhosis are of paramount importance [12, 13]. Timely recognition and efficacious interventions have the potential to reduce the likelihood of complications, enhance longevity, and improve the standard of living [14, 15]. Significantly, the current diagnosis of liver fibrosis predominantly hinges on Transient Elastography or hepatic biopsy, presenting persistent challenges in primary care contexts [16–19]. The exploration of noninvasive screening rooted in clinical manifestations continues to be a focal point of research within this domain.

Diabetic retinopathy (DR) is a severe ocular complication that affects patients with type 2 diabetes mellitus (T2DM) and has profound ramifications for global health [20–22]. Retinopathy manifests in two distinct forms: non-proliferative and proliferative [23]. Macular edema may manifest at any juncture in the disease trajectory, posing a formidable threat to visual acuity [24]. Furthermore, the prolonged duration of diabetes mellitus and suboptimal management of blood glucose and arterial blood pressure are the principal drivers of the onset and progression of retinopathy [24]. Recent studies have explored the robust correlation between retinopathy and significant liver fibrosis [25]. Specifically, evidence suggests that non-proliferative diabetic retinopathy (NPDR) may also be linked to liver fibrosis, as the underlying mechanisms of insulin resistance and metabolic dysregulation present in patients with NPDR can similarly affect liver health. Additionally, emerging evidence has highlighted a broader association between liver fibrosis and ocular manifestations in patients with diabetes, reinforcing the shared pathogenic mechanisms between these conditions [26–28]. Although the precise mechanisms remain elusive, shared pathogenic pathways have been identified in patients with T2DM and hepatic fibrosis who experience DR [29]. These pathways include insulin resistance, metabolic inflammation arising from disturbances in glucolipid metabolism, and oxidative stress [30–32]. Significant liver fibrosis may exacerbate systemic insulin resistance and hyperglycemia [33, 34], potentially contributing to the progression of retinopathy [35, 36]. This phenomenon is likely due to the augmentation of hepatic and systemic insulin resistance by liver fibrosis, the promotion of dyslipidemia, and the initiation of the synthesis of diverse pro-inflammatory mediators, which could contribute to the occurrence of chronic vascular complications of diabetes [37]. The evaluation of retinopathy severity involves retinal imaging and is typically classified into five grades: no diabetic retinopathy (NDR), mild non-proliferative diabetic retinopathy (NPDR), moderate NPDR, severe NPDR, and proliferative diabetic retinopathy (PDR) [38]. Recent studies have found that retinopathy may independently indicate significant liver fibrosis in a T2DM cohort [25].

The use of machine-learning (ML) methodologies in clinical research has steadily increased in recent years, propelled by the burgeoning availability of intricate clinical datasets [39]. This has garnered considerable interest and acclaim from a broad spectrum of clinicians and researchers. Unlike conventional statistical approaches, ML confers notable advantages in terms of prognostic accuracy and identification of latent patient subgroups characterized by distinct physiological profiles and prognostic trajectories [39]. Moreover, ML exhibits adeptness in navigating intricate interactions and nonlinear relationships, often beyond the purview of conventional clinical study methodologies, which are invariably influenced by multifaceted interrelationships among myriad factors [40, 41]. Consequently, an increasing number of clinical investigations have focused on adopting ML techniques [42]. The burgeoning interest in delineating the association between retinopathy and significant liver fibrosis underscores this frontier of inquiry. No previous studies have investigated predictive models to elucidate the risk of significant liver fibrosis in individuals with retinopathy. Thus, it is imperative to develop a predictive model that amalgamates diverse risk factors to appraise the propensity towards significant liver fibrosis onset in patients with retinopathy.

The primary objective of this investigation was to develop an interpretable ML framework, leveraging clinical data to predict the likelihood of significant liver fibrosis onset in patients with DR. Additionally, we endeavored to gauge the practical viability of such an interpretable ML model in clinical settings.

## Materials and methods

### Data sources

This study harnessed data sourced from the National Health and Nutrition Examination Survey (NHANES) repository, a publicly accessible online database renowned for its comprehensive, longitudinal, and multidimensional nature [43]. This invaluable resource facilitated the development of predictive models tailored specifically for individuals afflicted with retinopathy. Noteworthy for its representation and scope, the NHANES database furnishes vital insights essential for the formulation of nutrition and public health strategies. It is imperative to underscore that all participants enrolled in this database have provided explicit informed consent or have had proxies duly authorized to do so.

### Study population

**Fig. 1:**
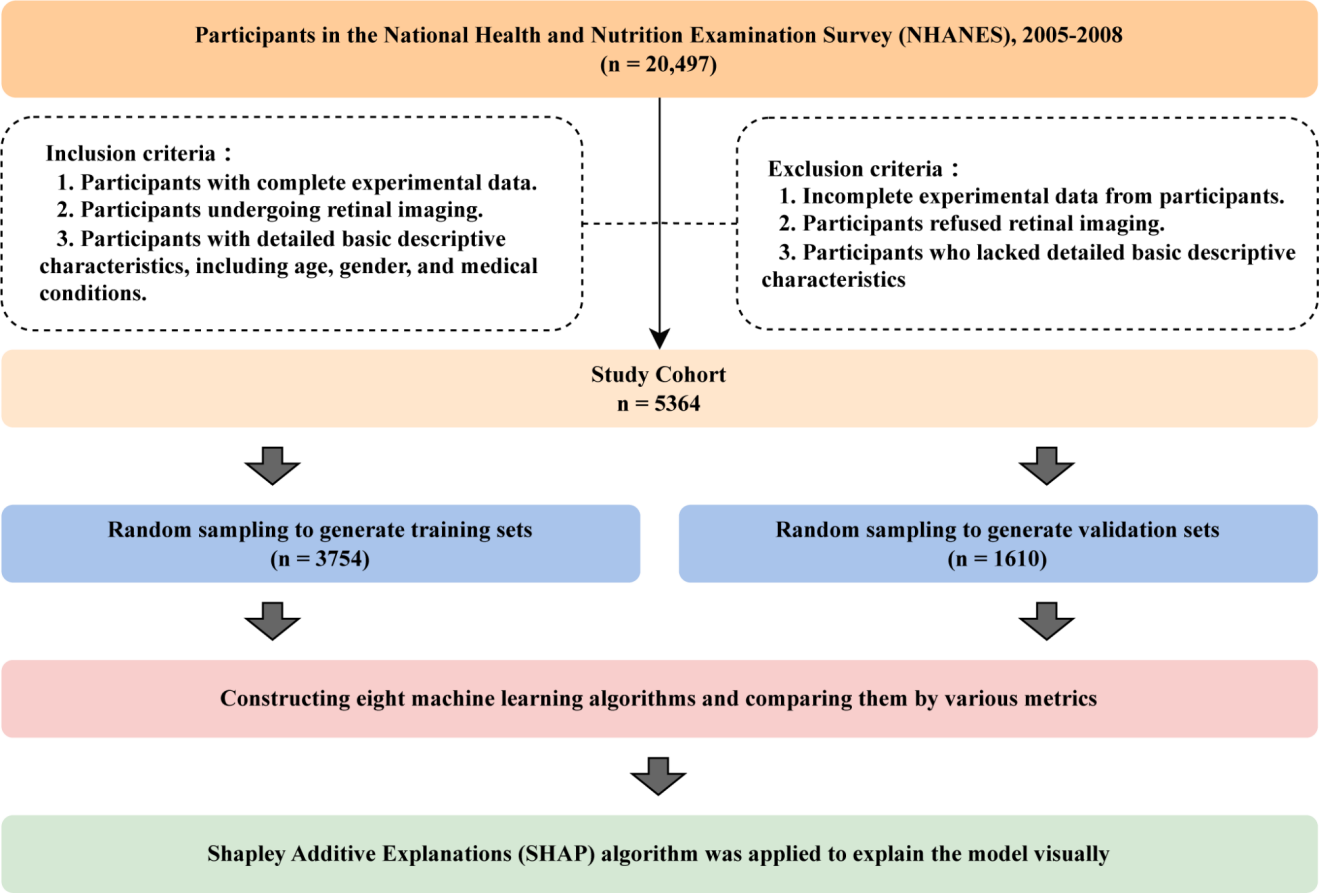
Flowchart of this study

The data used in this study were extracted from the NHANES database, covering the years 2005 to 2008. Our inclusion criteria were designed to ensure that participants possessed comprehensive experimental data, including key descriptors such as retinal imaging outcomes, age, gender, and relevant medical history. The exclusion criteria eliminated individuals who [1] had incomplete or missing experimental data [2], refused retinal imaging, or [3] lacked essential descriptors. These criteria represent additional screening and exclusions applied beyond the original NHANES study design, tailored specifically to meet the requirements of our analysis. We did not rely solely on NHANES’s initial selection process but performed further filtering to ensure the integrity and relevance of the data for our study. After this selection process, a total of 5,364 participants were included in the final analysis (Fig. 1).

### Retinopathy grading

Via meticulous retinal imaging assessments, we discerned the gradations of retinopathy severity, aligning them with the NHANES grading protocol [21]. Accordingly, we categorized retinopathy into four distinct tiers: Grade 1 denoting absence of retinopathy; Grade 2 indicating mild Non-proliferative Diabetic Retinopathy (NPDR); Grade 3 reflecting moderate to severe NPDR; and Grade 4 signifying retinopathy characterized by neovascularization at the optic disc (PR).

### Liver fibrosis classification

The Fibrosis-4 index (FIB-4) stands as a prominent non-invasive biomarker widely utilized in the assessment of advanced liver fibrosis [44]. Through the application of the FIB-4, we ascertain the degree of liver fibrosis. It has been established that the FIB-4 calculation, rooted in age, aspartate aminotransferase (AST), alanine aminotransferase (ALT), and platelet (PLT), serves as an effective tool in discerning various stages of liver fibrosis and identifying patients afflicted with significant fibrosis. The formula governing the computation of the FIB-4 index is as follows: FIB-4 index = age × AST (IU/L) / platelet count (× 10^9 /L) × √ALT (IU/L). This computation enables the classification of liver fibrosis into four distinct grades: FIB-4 < 1.45 signifies mild fibrosis (F0-F1), while 1.45–3.25 corresponds to moderate fibrosis (F2), and > 3.25 denotes advanced fibrosis/cirrhosis (F3-F4). In this investigation, lesions falling within the F0-F1 category were categorized as absent/mild fibrotic lesions, whereas those within the F2-F4 range were deemed indicative of significant liver fibrosis/cirrhosis [45].

### Data preprocessing and feature selection

The omission of datasets containing absent values in this study ensured the completeness of data from the included participants, obviating the necessity for employing interpolation methods. In the realm of feature selection, the Boruta algorithm emerges as a robust method, distinguished by its substantial stability in the identification and retention of relevant variables [46]. The core principle underlying this methodology involves a comparative analysis of the significance of actual predictive variables against artificially generated counterparts, termed ‘shadow variables,’ through a series of statistical evaluations and multiple iterations of Random Forest (RF) algorithms [47]. Subsequently, variables deemed non-essential, including all shadow variables, are systematically excised. Through the Boruta algorithm, this study ultimately included demographic characteristics encompassed age, sex, and race/ethnicity. Examination data comprised measurements of height (cm), weight (kg), and body mass index (BMI, kg/m²), while disease history documented hepatitis C virus(HCV) infection, T2DM, and DR.

### Machine learning models

We adopted a random sampling approach, selecting 70% of participants from the NHANES database spanning 2005 to 2008 for the training set, while allocating the remaining 30% for internal validation. Subsequently, we embarked on the construction and assessment of eight supervised ML models, encompassing diverse methodologies such as extreme gradient boosting (XGBoost), RF, multilayer perceptron (MLP), support vector machine (SVM), logistic regression (LR), Naive Bayes (NB), decision tree (DT), and K-nearest neighbor (KNN). To mitigate the risk of overfitting, each model underwent rigorous tenfold cross-validation. To gauge the alignment between clinical utility and predictive accuracy, we conducted Decision Curve Analysis (DCA). Furthermore, the reserved 30% of data facilitated internal validation, offering insights into the predictive model’s efficacy. Subsequently, leveraging the SHapley Additive exPlanations (SHAP) method, we discerned the most salient risk factors underpinning the propensity towards significant liver fibrosis. SHAP values provided a visual depiction elucidating the significance of each feature and its contribution to the risk of significant liver fibrosis [48]. Implementation of the SHAP methodology was executed using R, specifically leveraging SHAPVIZ version 0.9.3.

### Model performance evaluation

The delineation of the ML model’s efficacy is quantitatively assessed through the computation of several pivotal metrics: the area under the receiver operating characteristic curve (AUC), the optimal threshold, Accuracy, sensitivity, specificity, precision, F1 score, Brier score, and the confidence intervals (CI). Among these, the AUC and F1 score stand as paramount indicators for appraising the model’s performance [49, 50]. The AUC provides a comprehensive encapsulation of the ROC curve’s performance, furnishing a solitary figure that aggregates the classifier’s efficacy. This metric is particularly advantageous when juxtaposing various ROC curves—especially under circumstances where these curves may intersect—because it allows for the ranking of models based on their aggregate performance, thus simplifying the evaluation process. The F1 score emerges as a pivotal metric for further scrutiny of the ML models’ effectiveness. By amalgamating precision and recall, it offers a robust measure of accuracy particularly suited for handling datasets with imbalanced classes, thereby establishing a judicious equilibrium between these two elements. Precision measures the accuracy of positive predictions, while recall elucidates the model’s adeptness at identifying true positive instances, thereby mirroring the model’s sensitivity.

### Statistical analysis

Data conforming to a normal distribution were depicted as mean ± standard deviation (SD), while median and interquartile range (IQR) were employed to characterize non-normally distributed data. Categorical data were articulated in terms of frequencies and percentages. Continuous variables underwent analysis via the Wilcoxon rank sum test, whereas categorical variables were scrutinized using the chi-square test (Table 1). All *p*-values are two-sided, with *p*-values < 0.05 considered statistically significant. All statistical analyses were performed using SPSS software (Version 23.0), R software (Version 3.3.2), and Python software (Version 3.10.4).

**Table 1:** Demographic characteristics in populations with different degrees of liver fibrosis

Variables	Training set (*n* = 3754)				Validation set (*n* = 1610)		
	No/mild hepatic fibrosis	Significant liver fibrosis/cirrhosis			No/mild hepatic fibrosis	Significant liver fibrosis/cirrhosis	
	(*n* = 2282)	(*n* = 1472)	*P*-value		(*n* = 966)	(*n* = 644)	*P*-value
**Baseline information**							
Age (years)	52.00 [46.00, 60.00]	70.00 [62.00, 78.00]	< 0.001***		52.00 [45.00, 61.00]	70.00 [62.00, 76.00]	< 0.001***
Gender [% (n)]							
Female	1245 (54.6)	621 (42.2)	< 0.001***		533 (55.2)	272 (42.2)	< 0.001***
Male	1037 (45.4)	851 (57.8)			433 (44.8)	372 (57.8)	
Race [% (n)]							
Non-Hispanic white	397 (17.4)	172 (11.7)	< 0.001***		205 (21.2)	79 (12.3)	< 0.001***
Non-Hispanic black	190 (8.3)	75 (5.1)			77 (8.0)	34 (5.3)	
Mexican American	1135 (49.7)	909 (61.8)			472 (48.9)	391 (60.7)	
Other Hispanic	470 (20.6)	287 (19.5)			177 (18.3)	120 (18.6)	
Other race	90 (3.9)	29 (2.0)			35 (3.6)	20 (3.1)	
**Physical examination**							
Weight (kg)	81.20 [69.20, 95.18]	78.15 [67.27, 89.70]	< 0.001***		80.30 [68.73, 93.70]	78.00 [65.95, 90.32]	0.001***
Height (cm)	166.70 [159.90, 174.40]	168.20 [160.20, 175.02]	0.072		166.65 [159.62, 173.70]	167.40 [160.20, 175.62]	0.105
BMI (kg/m^2^)	28.96 [25.08, 33.40]	27.66 [24.54, 31.39]	< 0.001***		28.80 [25.23, 33.05]	27.52 [24.50, 31.10]	< 0.001***
**Clinical information**							
PLT (10^9^/L)	40.00 [21.00, 283.00]	31.50 [19.00, 205.25]	< 0.001***		36.00 [20.00, 284.75]	37.00 [21.00, 209.00]	< 0.001***
ALT (U/L)	23.00 [19.00, 27.00]	24.00 [20.00, 31.00]	< 0.001***		22.00 [19.00, 27.00]	24.00 [20.00, 30.00]	< 0.001***
AST (U/L)	215.00 [23.00, 288.00]	176.00 [28.00, 229.00]	< 0.001***		224.00 [23.00, 297.75]	170.00 [27.00, 231.00]	< 0.001***
Retinopathy [% (n)]			< 0.005**				0.048*
No retinopathy (Level 1)	2032 (89.0)	1261 (85.7)			861 (89.1)	545 (84.6)	
Mild NPDR (Level 2)	199 (8.7)	178 (12.1)			83 (8.6)	83 (12.9)	
Moderate/severe NPDR (Level 3)	43 (1.9)	24 (1.6)			17 (1.8)	12 (1.9)	
PR (Level 4)	8 (0.4)	9 (0.6)			5 (0.5)	4 (0.6)	
Hepatitis C virus infection [% (n)]			0.421				0.238
Yes	57 (2.5)	44 (3.0)			21 (2.2)	21 (3.3)	
No	2225 (97.5)	1428 (97.0)			945 (97.8)	623 (96.7)	
T2DM [% (n)]			< 0.001***				0.120
Yes	405 (17.7)	366 (24.9)			184 (19.0)	144 (22.4)	
No	1877 (82.3)	1106 (75.1)			782 (81.0)	500 (77.6)	

Note: Data are presented as number (percentage) or median [IQR]

Abbreviations: BMI: body mass index; PLT: platelet count; ALT: alanine aminotransferase; AST: aspartate aminotransferase; T2DM: type 2 diabetes mellitus

## Results

### Demographic characteristics of participants

In accordance with the specified inclusion and exclusion criteria, a cohort of 5,364 individuals was recruited for this study and subsequently partitioned into a training set (*n* = 3754) and a validation set (*n* = 1,610) in a 7:3 ratio (Fig. 1). Within the training set, 1,472 participants (39.2%) presented with significant liver fibrosis/cirrhosis, mirroring the figures observed in the validation set, wherein 644 participants (40.0%) exhibited similar hepatic conditions. The median age (IQR) of individuals manifesting significant liver fibrosis/cirrhosis stood at 70 (62.00–78.00) years, a noteworthy contrast to those who did not exhibit mild fibrotic lesions (52.00 [46.00–60.00] years, *P* < 0.001). The distributions of Height, and HCV characteristics exhibited no discernible discrepancies between the training and validation sets. Sex, race, Weight, BMI, PLT, ALT, AST, Retinopathy, and T2DM have emerged as factors intricately linked to the progression of significant liver fibrosis. Remarkably, the absence of any disparity in T2DM prevalence within the validation set suggests potential disparities in data distribution between the two sets. Moreover, participants afflicted with retinopathy displayed heightened vulnerability to significant liver fibrosis development compared to their counterparts without retinopathy, a trend observed in both the training and validation sets (*P* < 0.05) (Table 1).

### Feature selection

**Fig. 2:**
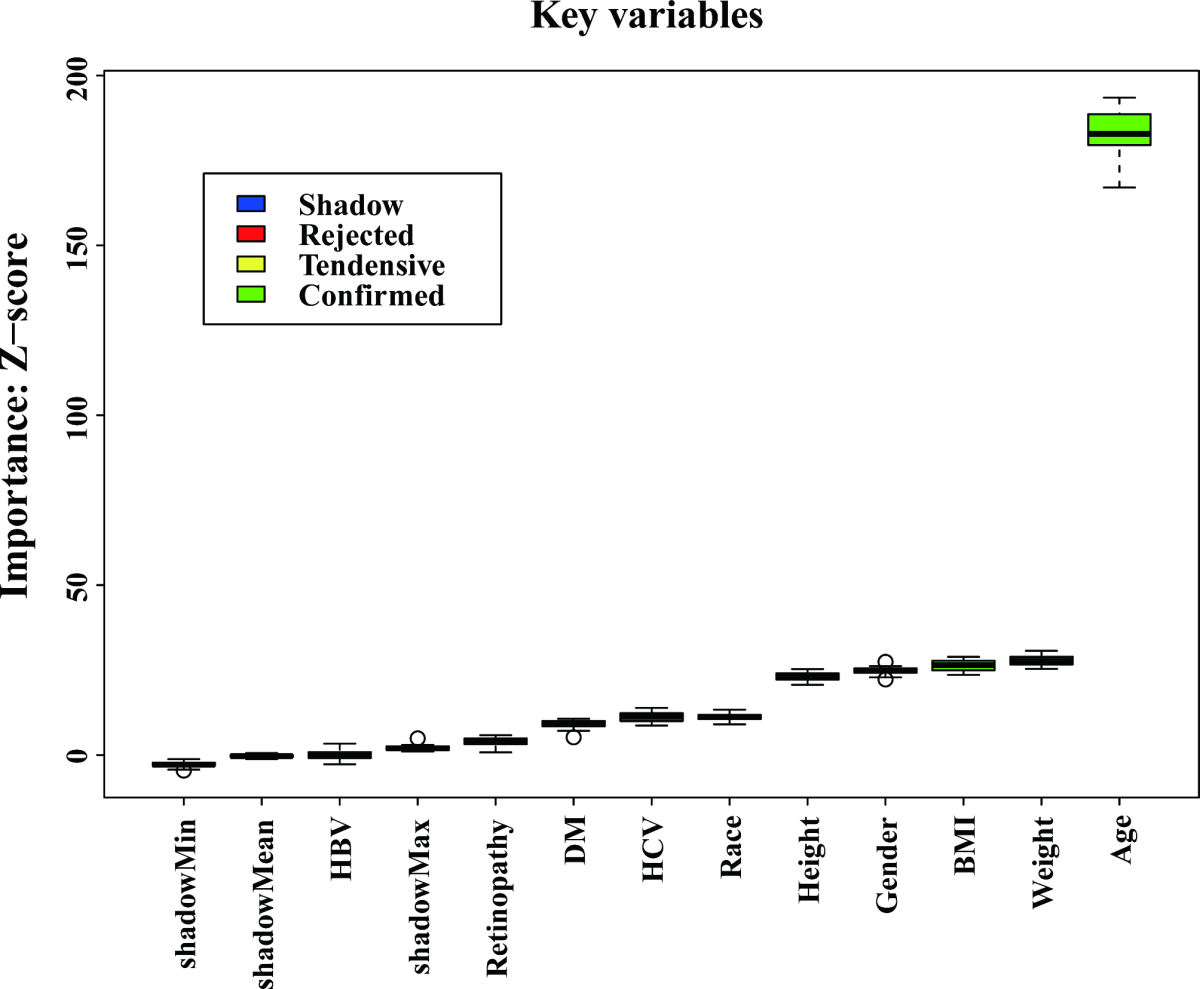
Feature selection based on the Boruta algorithm

AST, PLT, and ALT were excluded from the analysis prior to the Boruta algorithm due to their role in calculating the FIB-4 index. Including them would introduce redundancy and multicollinearity, which would distort the model’s accuracy and violate key assumptions of statistical modeling. Following their exclusion, the Boruta algorithm identified 9 variables with the strongest association with significant liver fibrosis (Fig. 2).

Among these, age was retained in the model despite being a component of FIB-4. This decision was made based on the independent and well-established clinical relevance of age in liver fibrosis progression. Age has been consistently recognized as a key determinant of liver disease severity due to its association with the cumulative exposure to various risk factors (e.g., metabolic disorders, viral hepatitis) and the declining regenerative capacity of the liver with advancing age. Moreover, age has been shown to modulate immune response and influence fibrosis development through age-related inflammatory processes and cellular senescence, which are independent of the specific contributions captured by the FIB-4 index.

By excluding age, the model would risk underestimating the impact of time-dependent factors on liver fibrosis, which are critical for accurate patient stratification and prognosis. Thus, while AST, PLT, and ALT were excluded to avoid circularity, age remains an indispensable variable due to its direct clinical implications beyond the scope of its role in FIB-4 calculation. As a result, 9 variables were selected for the final ML model development.

### Model evaluation

**Fig. 3:**
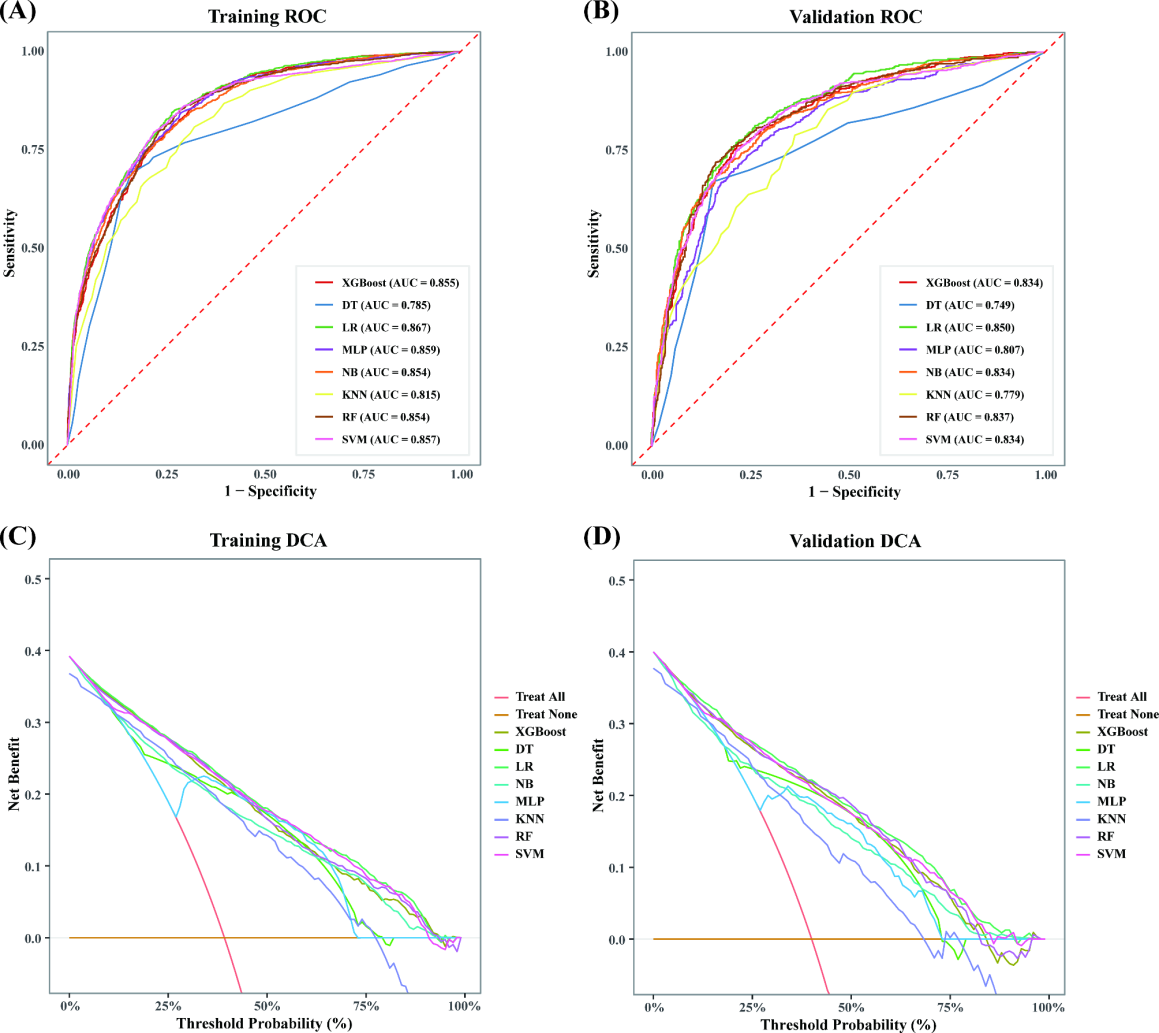
Prediction performance by AUROC for the eight machine-learning models in the training cohort (**A**) and validation cohort (**B**), along with the Decision Curve Analysis (DCA) curve in the training cohort (**C**) and validation cohort (**D**)

The LR algorithm emerged with the highest AUC of 0.867 (95% CI: 0.855–0.878) among the ML models employed for significant liver fibrosis prediction (Fig. 3A; Table 2). Notably, the LR model garnered a noteworthy F1 score of 0.749 (95% CI: 0.732–0.767). In the validation cohort, both the LR and RF models displayed the highest AUC values among the eight ML models (LR: 0.850, 95% CI: 0.829–0.869; RF: 0.837, 95% CI: 0.817–0.857) alongside respectable F1 scores (LR: 0.736, 95% CI: 0.709–0.763; RF: 0.732, 95% CI: 0.704–0.761) (Fig. 3B; Table 3). To further illustrate the classification performance of the LR model, confusion matrices were plotted for both the training and validation sets (Figures S1A and S1B, respectively). These matrices provide a granular view of the model’s true positives, false positives, true negatives, and false negatives. The results demonstrated that the LR model correctly classified a high number of true positives and true negatives, reinforcing its ability to accurately distinguish between patients with and without significant liver fibrosis. Importantly, the relatively low rate of false positives and false negatives underscores the model’s reliability in clinical risk prediction, minimizing the potential for both over-diagnosis and missed diagnoses. This analysis adds further weight to the LR model’s favorable performance metrics, complementing the high AUC and F1 scores. These findings affirm the LR model’s robustness and suitability for real-world clinical implementation. Furthermore, the pairwise comparisons of the AUCs between Logistic Regression and other models, as presented in Table S1, provide additional evidence of the statistical significance of these performance differences.

**Table 2:** Predictive performance of eight machine learning models on training sets

Model	AUC	Optimal cutoff	Accuracy	Sensitivity	Specificity	Precision	F1 score	Brier score
LR	0.867 (0.855, 0.878)	0.322	0.778 (0.765, 0.792)	0.845 (0.826, 0.863)	0.735 (0.717, 0.754)	0.673 (0.651, 0.695)	0.749 (0.732, 0.767)	0.144 (0.136, 0.150)
MLP	0.859 (0.847, 0.871)	0.405	0.764 (0.751, 0.778)	0.845 (0.826, 0.864)	0.712 (0.693, 0.731)	0.654 (0.632, 0.676)	0.737 (0.720, 0.754)	0.173 (0.169, 0.177)
SVM	0.857 (0.845, 0.869)	0.326	0.786 (0.773, 0.799)	0.795 (0.776, 0.815)	0.780 (0.763, 0.796)	0.700 (0.677, 0.722)	0.744 (0.727, 0.761)	0.148 (0.141, 0.155)
NB	0.854 (0.842, 0.866)	0.204	0.772 (0.759, 0.786)	0.771 (0.750, 0.794)	0.773 (0.755, 0.791)	0.687 (0.663, 0.710)	0.727 (0.708, 0.746)	0.169 (0.160, 0.177)
XGBoost	0.855 (0.843, 0.867)	0.385	0.769 (0.755, 0.783)	0.796 (0.775, 0.818)	0.751 (0.734, 0.770)	0.674 (0.652, 0.696)	0.730 (0.713, 0.747)	0.151 (0.144, 0.158)
RF	0.854 (0.842, 0.867)	0.399	0.780 (0.766, 0.793)	0.799 (0.778, 0.819)	0.767 (0.750, 0.786)	0.689 (0.666, 0.712)	0.740 (0.723, 0.757)	0.150 (0.143, 0.157)
KNN	0.815 (0.801, 0.828)	0.265	0.728 (0.714, 0.742)	0.808 (0.789, 0.828)	0.677 (0.658, 0.695)	0.617 (0.594, 0.638)	0.700 (0.682, 0.717)	0.183 (0.174, 0.192)
DT	0.785 (0.766, 0.792)	0.477	0.779 (0.729, 0.774)	0.693 (0.668, 0.719)	0.835 (0.819, 0.850)	0.731 (0.709, 0.753)	0.712 (0.692, 0.730)	0.168 (0.161, 0.176)

Abbreviations: XGBoost: Extreme Gradient Boosting; RF: Random Forest; MLP: Multilayer Perceptron; SVM: Support Vector Machine; LR: Logistic Regression; NB: Naive Bayes; DT: Decision Tree; KNN: K-Nearest Neighbors; AUC: Area Under the Curve

**Table 3:** Predictive performance of eight machine learning models on the validation set

Model	AUC	Optimal cutoff	Accuracy	Sensitivity	Specificity	Precision	F1 score	Brier score
LR	0.850 (0.829, 0.869)	0.409	0.779 (0.758, 0.799)	0.770 (0.734, 0.803)	0.785 (0.759, 0.810)	0.704 (0.671, 0.740)	0.736 (0.709, 0.763)	0.153 (0.143, 0.164)
RF	0.837 (0.817, 0.857)	0.478	0.790 (0.771, 0.810)	0.721 (0.686, 0.754)	0.836 (0.812, 0.859)	0.746 (0.712, 0.780)	0.732 (0.704, 0.761)	0.158 (0.148, 0.169)
NB	0.834 (0.813, 0.854)	0.209	0.770 (0.749, 0.791)	0.721 (0.687, 0.755)	0.802 (0.776, 0.827)	0.708 (0.673, 0.743)	0.714 (0.688, 0.742)	0.158 (0.148, 0.169)
SVM	0.834 (0.813, 0.856)	0.388	0.770 (0.750, 0.790)	0.750 (0.715, 0.783)	0.784 (0.758, 0.810)	0.698 (0.663, 0.730)	0.723 (0.695, 0.749)	0.159 (0.149, 0.171)
XGBoost	0.834 (0.814, 0.855)	0.387	0.775 (0.754, 0.794)	0.761 (0.728, 0.792)	0.784 (0.758, 0.809)	0.702 (0.666, 0.736)	0.730 (0.704, 0.756)	0.161 (0.149, 0.173)
MLP	0.807 (0.784, 0.828)	0.448	0.752 (0.730, 0.773)	0.720 (0.685, 0.754)	0.774 (0.746, 0.800)	0.680 (0.642, 0.716)	0.699 (0.669, 0.725)	0.186 (0.180, 0.192)
KNN	0.779 (0.757, 0.802)	0.265	0.697 (0.672, 0.720)	0.789 (0.755, 0.821)	0.635 (0.603, 0.668)	0.590 (0.553, 0.625)	0.675 (0.644, 0.703)	0.205 (0.192, 0.219)
DT	0.749 (0.725, 0.774)	0.473	0.774 (0.754, 0.794)	0.670 (0.635, 0.709)	0.843 (0.819, 0.866)	0.740 (0.706, 0.774)	0.703 (0.675, 0.733)	0.175 (0.163, 0.186)

Abbreviations: XGBoost: Extreme Gradient Boosting; RF: Random Forest; MLP: Multilayer Perceptron; SVM: Support Vector Machine; LR: Logistic Regression; NB: Naive Bayes; DT: Decision Tree; KNN: K-Nearest Neighbors; AUC: Area Under the Curve

**Fig. 4:**
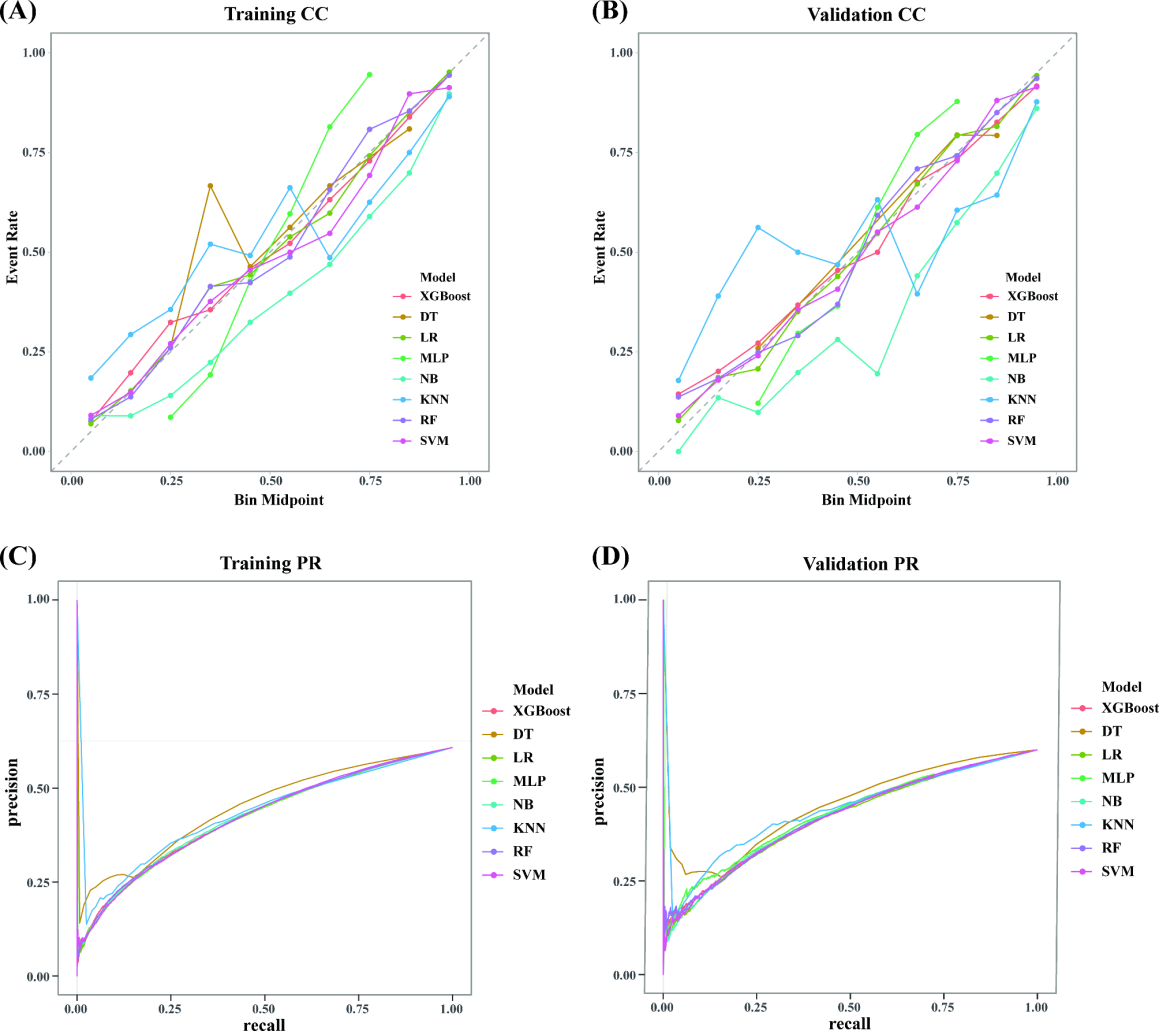
(**A**) Training cohort Calibration curves; (**B**) Validation cohort Calibration curves; (**C**) Training cohort Precision-Recall Curve; (**D**) Validation cohort Precision-Recall Curve

Detailed DCA of the training dataset revealed that the LR model exhibited excellent performance among the ML paradigms, affirming its robust efficacy in clinical implementation (Fig. 3C). Concurrently, the DCA results for the validation sets confirm that the use of the LR model for risk prediction leads to substantial positive net benefits (Fig. 3D). Furthermore, Panels A, and B of Fig. 4 depict the calibration curves for various models in the training, and validation sets, respectively. The LR model exhibits good calibration on all datasets, and the calibration curves are highly coincident with the ideal 45-degree baseline, indicating that the match between its predicted event rates and the actual event rates is more accurate. In contrast, the KNN and MLP models exhibit significant calibration bias on both the internal and external validation sets, especially in the higher probability intervals, where their predictions deviate more from the actual observations.

Moreover, the analysis of precision-recall (PR) curves across the models (Fig. 4C-D) further highlights the superior performance of the LR model. PR curves are especially informative when evaluating models on imbalanced datasets, as they focus on the balance between precision (positive predictive value) and recall (sensitivity). The LR model demonstrates high precision and recall, reflecting its ability to minimize false positives while maintaining a strong sensitivity to true positives. These results underscore the LR model’s superior discriminatory power and reliability for clinical risk prediction in imbalanced clinical datasets, making it the optimal choice for this application.

### Visualization by SHAP

**Fig. 5:**
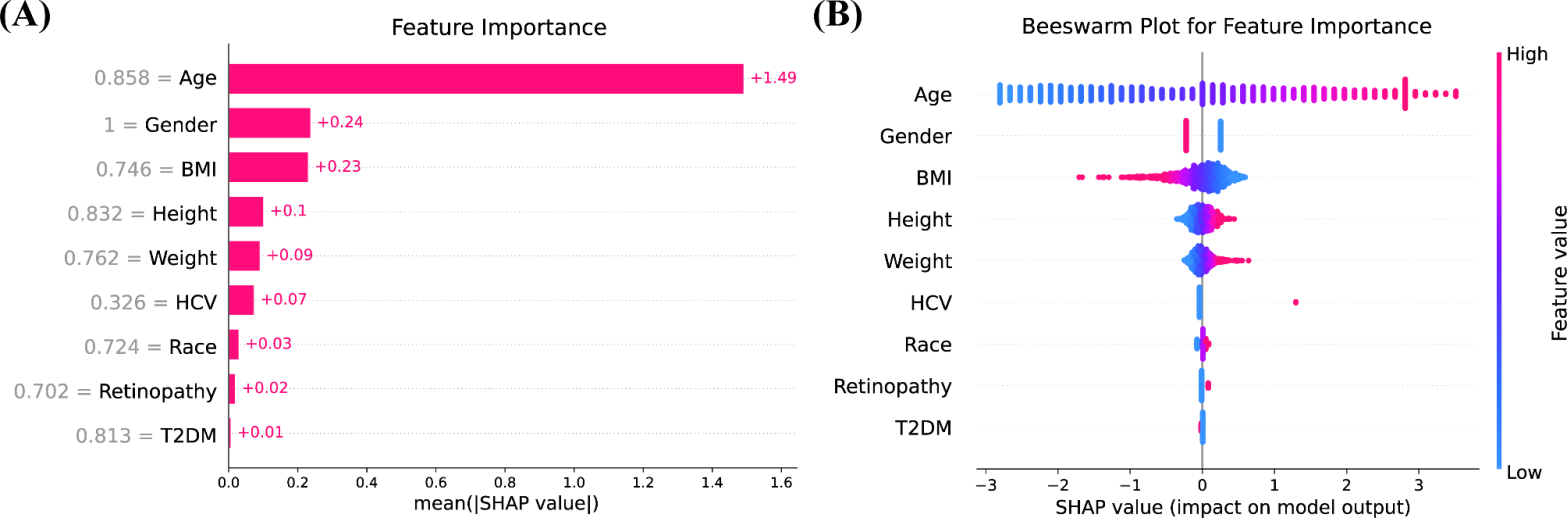
(**A**) SHapley Additive exPlanations (SHAP) feature importance shown according to the mean absolute SHAP value of each feature; (**B**) SHAP summary plot showing the distribution of the SHAP values of each feature

Figure 5A illustrates the importance of the SHAP features for the LR model. The features under scrutiny were arranged in descending order of their influence on the projected outcomes, as indicated by the mean absolute value of SHAP. Among these, the top five pivotal features were alterations in age, gender, BMI, height, and weight. The SHAP summary plot of the LR model revealed the effects of these features on the prognostic model (Fig. 5B). Within the predictive framework, elevated SHAP values associated with specific features indicate an augmented predisposition to significant liver fibrosis. For instance, older individuals exhibit heightened susceptibility to significant liver fibrosis compared to their younger counterparts.

### Interpreting machine learning models at the patient level

We employed the SHAP method to elucidate the individual predictions for the patients and evaluated the impact of the LR model on individual patient features (Figure S2). The contribution of each feature is depicted in color, with red indicating a positive contribution and blue indicating a negative contribution. The length of the color bar represents the magnitude of the contribution. For patient A (classified in the “true positive” group), the LR model indicated a higher likelihood of significant liver fibrosis. Conversely, for patient B (classified in the “true negative” group), the LR model inferred a relatively low probability of developing significant liver fibrosis.

### Construction and clinical application of an online prediction tool

Based on a previously constructed logistic regression model, we successfully developed an online calculator (https://lalalaanjila.shinyapps.io/Logistics_app/) for predicting the probability of a patient experiencing significant liver fibrosis. The calculator integrates several clinical variables and transforms the input individual data into the predicted probability of significant liver fibrosis risk through the Logit transform formula, thus providing clinicians with a convenient and accurate platform for individualized risk assessment (Figure S3). Compared with traditional scoring systems, this calculator not only improves the accuracy of risk prediction, but also significantly simplifies the clinical process, allowing individualized treatment decisions to be made more efficiently. Preliminary validation showed that the tool performed on a validation set in line with the original expectations of the model, further supporting its potential application value in clinical practice.

## Discussion

The principal aim of this investigation was to craft interpretable ML models using clinical data to predict the likelihood of significant liver fibrosis in patients with DR. Our primary dataset was sourced from the NHANES database, a comprehensive repository renowned for its extensive utilization across diverse research domains in recent years. In our study, we found a positive correlation between the severity of DR and significant liver fibrosis. Both retinopathy and liver fibrosis pose significant global health challenges, often manifesting subtly, with many patients exhibiting no overt symptoms [51–53]. These two maladies are intertwined, elevating the risk of cirrhosis and cancer development in individuals with DR [54]. Consequently, there is a pressing need for a user-friendly, non-invasive modality for early stage liver fibrosis detection in patients with DR. We investigated the relationship between retinopathy and significant liver fibrosis, and our findings indicate that retinopathy could serve as a pivotal indicator of significant liver fibrosis progression in diabetic cohorts.

In the clinical setting, the coexistence of DR and significant liver fibrosis often manifests subtly, with many patients displaying no overt symptoms, making the simultaneous management of these conditions challenging [51]. Given the prevalence of T2DM and its complications, it is critical to recognize the often-overlooked progression of liver diseases, such as significant liver fibrosis, which may lead to compensatory or decompensatory chronic liver diseases [54]. Unfortunately, due to the generally asymptomatic nature of liver fibrosis in its early stages, patients with DR or T2DM frequently underestimate the severity or presence of liver conditions. This recognition underscores the urgent need for innovative approaches in clinical practice to facilitate the early detection and management of liver fibrosis among these patients. Our study advocates the integration of routine liver health assessments into diabetes care protocols by leveraging common clinical features and biomarkers to construct predictive models. Such models can stratify patients with DR according to the risk of liver fibrosis, enabling targeted early screening, prevention, and intervention strategies. This proactive approach aims not only to manage retinopathy, but also to preempt and mitigate the progression of liver fibrosis, thereby improving overall patient outcomes and survival rates.

This study harnessed the power of ML algorithms to predict the risk of significant liver fibrosis in individuals with DR. We meticulously scrutinized eight distinct ML prognostic methodologies, unveiling the LR model as the frontrunner, boasting the highest AUC of 0.867 (95% CI: 0.855–0.878), coupled with an impressive F1 score of 0.749 (95% CI: 0.732–0.767). To verify the validity and applicability of our findings, we meticulously validated the LR model against a validation dataset. In this independent validation cohort, the LR model once again eclipsed its ML counterparts, registering an AUC of 0.850 and an F1 score of 0.736. Furthermore, to unravel the intricacies of our LR model, we employed SHAP summaries and dependency plots to reveal the principal predictors of significant liver fibrosis in the DR population. The elucidation provided by the SHAP imparts significance to clinical metrics that are readily assessable in practice, such as age, gender, BMI, height, and weight, all of which are deemed pivotal features in the ML framework for significant liver fibrosis prognosis. Additionally, the presence of DR emerged as a noteworthy factor in the LR model, further amplifying its prognostic value.

Furthermore, the online calculator we developed provides an accessible, user-friendly tool that fits seamlessly into clinical workflows, potentially allowing for broader adoption in outpatient settings without the need for complex testing. Predicting liver fibrosis risk in DR patients has implications for both hepatologic and ophthalmologic management, as early fibrosis identification facilitates timely intervention, possibly mitigating further progression of fibrosis and related comorbidities [55, 56]. By bridging these clinical domains, this model aligns with personalized medicine principles, offering a streamlined pathway to integrate hepatic risk management within the care continuum for diabetic retinopathy patients.

Numerous studies have investigated the relationship between DR and significant liver fibrosis [25, 29, 57]. Although the precise mechanism remains elusive, a close correlation has been identified. These connections include insulin resistance, inflammation stemming from glucolipid metabolic disorders, and oxidative stress [37]. Studies have indicated that liver fibrosis can exacerbate retinopathy by intensifying systemic insulin resistance and hyperglycemia [37]. Furthermore, mounting evidence suggests that dysregulation of the microbiome and its metabolites may foster the onset and progression of hepatocellular steatosis, inflammation, and fibrosis in non-alcoholic fatty liver disease and DR [58, 59]. Cumulatively, the robust correlation between DR and significant liver fibrosis suggests that DR could serve as a clinical biomarker for the advancement of substantial liver fibrosis. For the first time, we harnessed the power of ML to elucidate the precise incidence of significant liver fibrosis in patients with DR, culminating in a gratifying outcome. This study highlighted the importance of screening for hepatic ailments in patients with diabetes. In particular, we emphasize the importance of early screening and treatment of liver disease in patients with retinopathy who present to the eye clinic of a healthcare facility with ocular discomfort. This endeavor not only holds promise for ameliorating anticipated survival rates, but also provides patients with superior avenues for health stewardship. Moreover, we advocate the use of transient elastography to corroborate the diagnoses in patients with retinopathy.

The FIB-4 index offers a convenient, non-invasive means to assess liver fibrosis, but it does have limitations. Initially validated for chronic hepatitis C, FIB-4’s performance can vary across different populations, such as patients with diabetes or obesity, where the progression and nature of fibrosis may be influenced by multiple factors [60, 61]. Furthermore, while histopathology and elastography techniques (like transient or magnetic resonance elastography) provide detailed, quantitative insights into liver stiffness [62], FIB-4 gives an estimation rather than a direct measure, which may lead to misclassification, especially in patients with intermediate fibrosis stages [63]. Nevertheless, FIB-4’s accessibility and ease of use make it a valuable screening tool in settings where advanced imaging is unavailable.

In our study, FIB-4 was chosen for its proven utility in large-scale, population-based assessments, allowing us to integrate it into a machine learning framework that incorporates additional clinical variables for a comprehensive evaluation. By leveraging this approach, we provide a method to enhance risk stratification in diabetic retinopathy patients, where liver fibrosis is a critical concern, underscoring the practical value of a multimodal predictive model.

Despite the robust sample size of this study and the alignment of the outcomes with our hypotheses, several limitations persisted, necessitating further refinement. First, the utilization of the NHANES database, which is monocentric, for training and testing our ML model may introduce racial bias, constraining its generalizability across diverse populations. To enhance the model applicability, inclusive datasets from various sources are imperative for comprehensive training and validation. Second, given the extraction of data from publicly available databases, the inherent bias stemming from missing data is unavoidable. While efforts have been made to mitigate its impact, missing residual information or bias may still persist. Third, due to its retrospective nature, this study unavoidably had a selection bias. Leveraging data solely from a single NHANES database underscores the necessity for multicenter, large-scale clinical investigations. Fourth, reliance on serological markers exclusively to construct a liver fibrosis severity model, without biopsy or FibroScan diagnostics, poses a limitation in accurately gauging the extent of fibrosis. Future studies could explore the amalgamation of diverse diagnostic modalities for a more nuanced and dependable fibrosis assessment. Prospective studies are warranted to investigate the plausible causal nexus between retinopathy and T2DM-associated liver fibrosis.

## Conclusion

ML models employing readily available clinical data can identify patients with DR who are prone to significant liver fibrosis. The SHAP methodology facilitates the interpretation of the ML model predictions, rendering them comprehensible for clinical implementation. In addition, doctors can intervene early and reduce the risk of complications before patients develop serious conditions such as significant liver fibrosis and cirrhosis.

## Electronic supplementary material

Below is the link to the electronic supplementary material.

Supplementary Material 1

Supplementary Material 2

Supplementary Material 3

Supplementary Material 4

Supplementary Material 5

## Data Availability

The datasets generated and/or analyzed during the current study are available in the GitHub repository, accessible via the following link: https://github.com/lalalaanjila/NHANES_Data_Analysis.git.

## Abbreviations

ALT: Alanine Aminotransferase
AST: Aspartate Aminotransferase
AUC: Area Under the Receiver Operating Characteristic Curve
BMI: Body Mass Index
CI: Confidence Intervals
DCA: Decision Curve Analysis
DR: Diabetic Retinopathy
DT: Decision Tree
FIB-4: The Fibrosis-4 Index
HCV: Hepatitis C Virus
IQR: Interquartile Range
KNN: K-Nearest Neighbor
LR: Logistic Regression
ML: Machine Learning
MLP: Multilayer Perceptron
NB: Naive Bayes
NHANES: National Health and Nutrition Examination Survey
NPDR: Non-proliferative Diabetic Retinopathy
PDR: Proliferative Diabetic Retinopathy
PLT: Platelet
RF: Random Forest
SD: Standard Deviation
SHAP: SHapley Additive exPlanations
SVM: Support Vector Machine
T2DM: Type 2 Diabetes Mellitus
XGBoost: Extreme Gradient Boosting
