# Supplementary figures and images for: Explainable machine learning model for predicting the risk of significant liver fibrosis in patients with diabetic retinopathy
Source: BMC Med Inform Decis Mak. 2024 Nov 11;24:332. doi: 10.1186/s12911-024-02749-z (PMC11552118; doi:10.1186/s12911-024-02749-z)

**Figure S1.** (A) Training set confusion matrix; (B) Validation set confusion matrix.


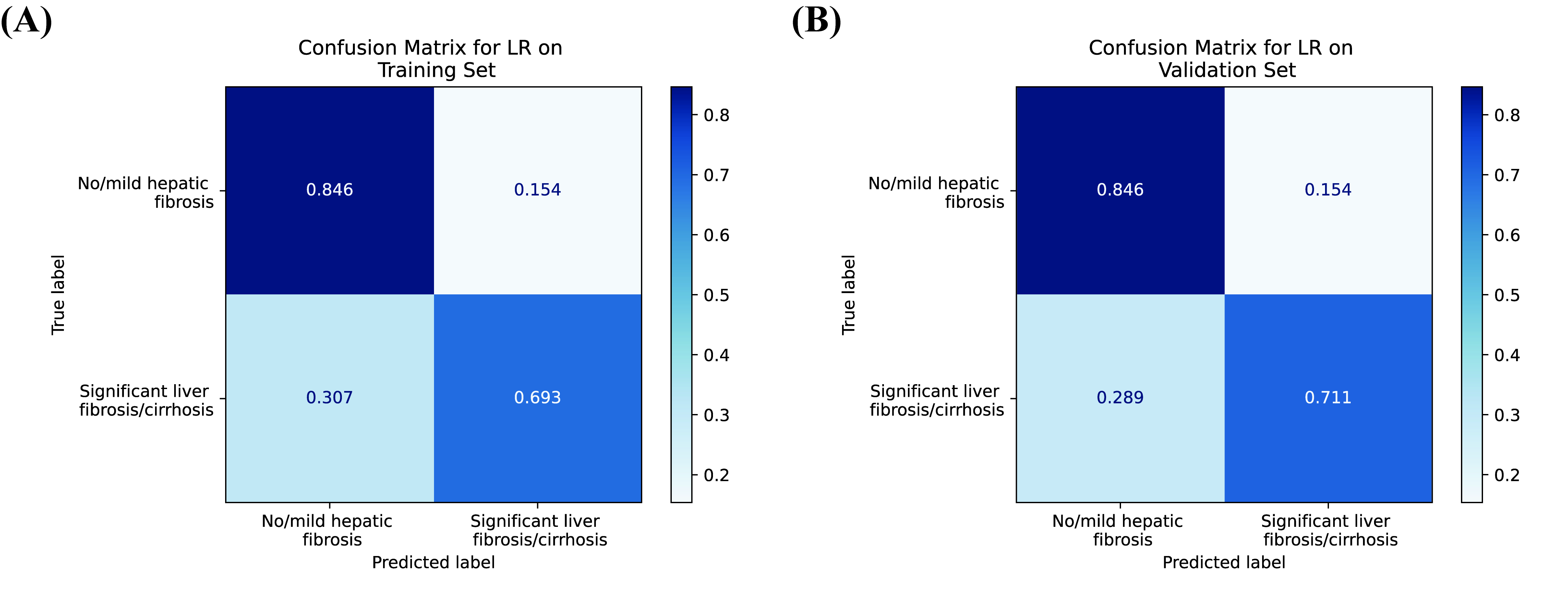

Supplement: Supplementary file 1 — Supplementary Material 1 [file 12911_2024_2749_MOESM1_ESM.docx]

**Figure S3.** A web predictor for predicting significant liver fibrosis.


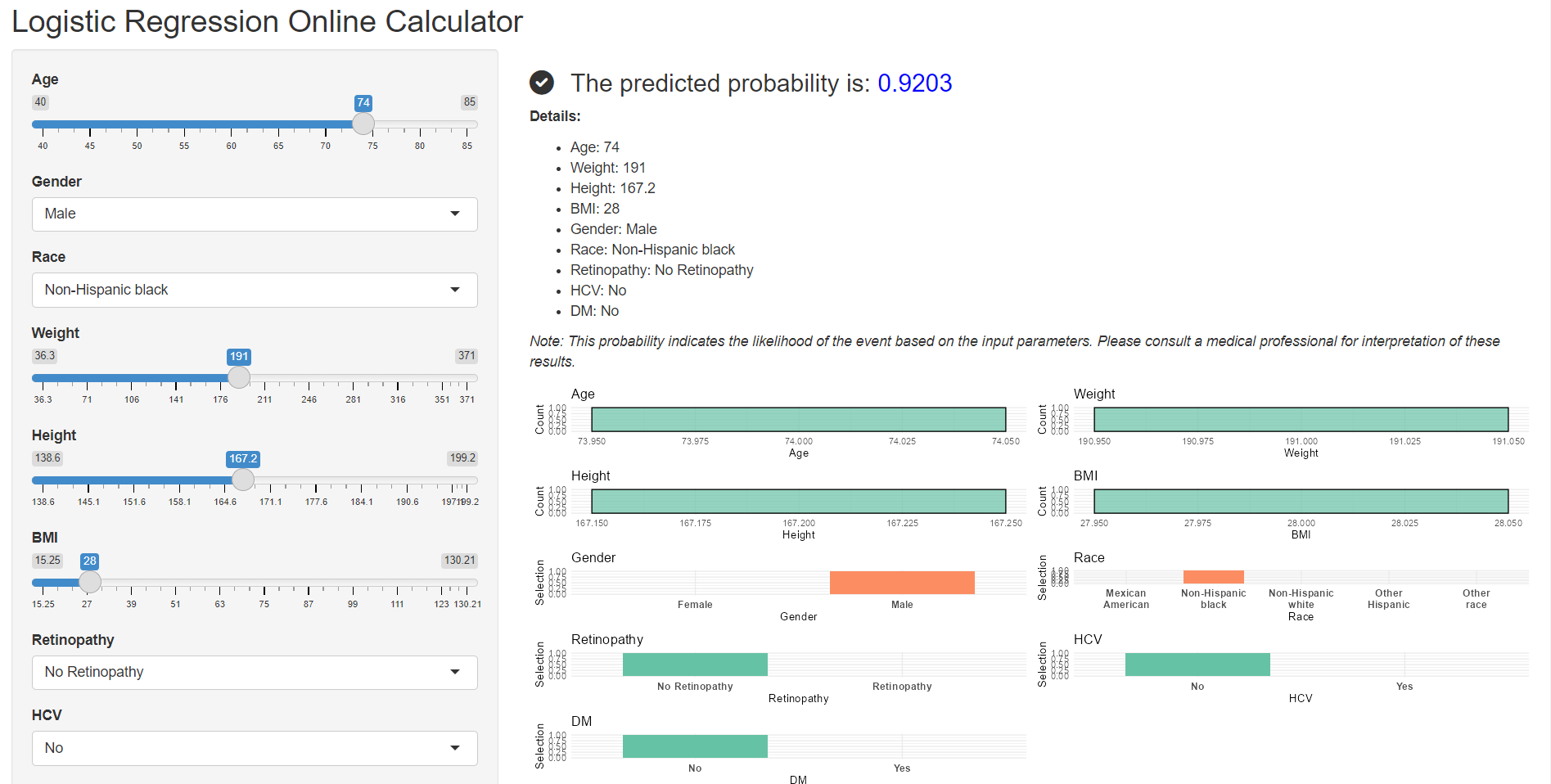

Supplement: Supplementary file 3 — Supplementary Material 3 [file 12911_2024_2749_MOESM3_ESM.docx]
