# Supplementary material for: Explainable machine learning model for predicting the risk of significant liver fibrosis in patients with diabetic retinopathy
Source: BMC Med Inform Decis Mak. 2024 Nov 11;24:332. doi: 10.1186/s12911-024-02749-z (PMC11552118; doi:10.1186/s12911-024-02749-z)

**Figure S2.** SHapley Additive exPlanations (SHAP) force plot of (A) Patient A (true positive) and (B) Patient B (true negative).


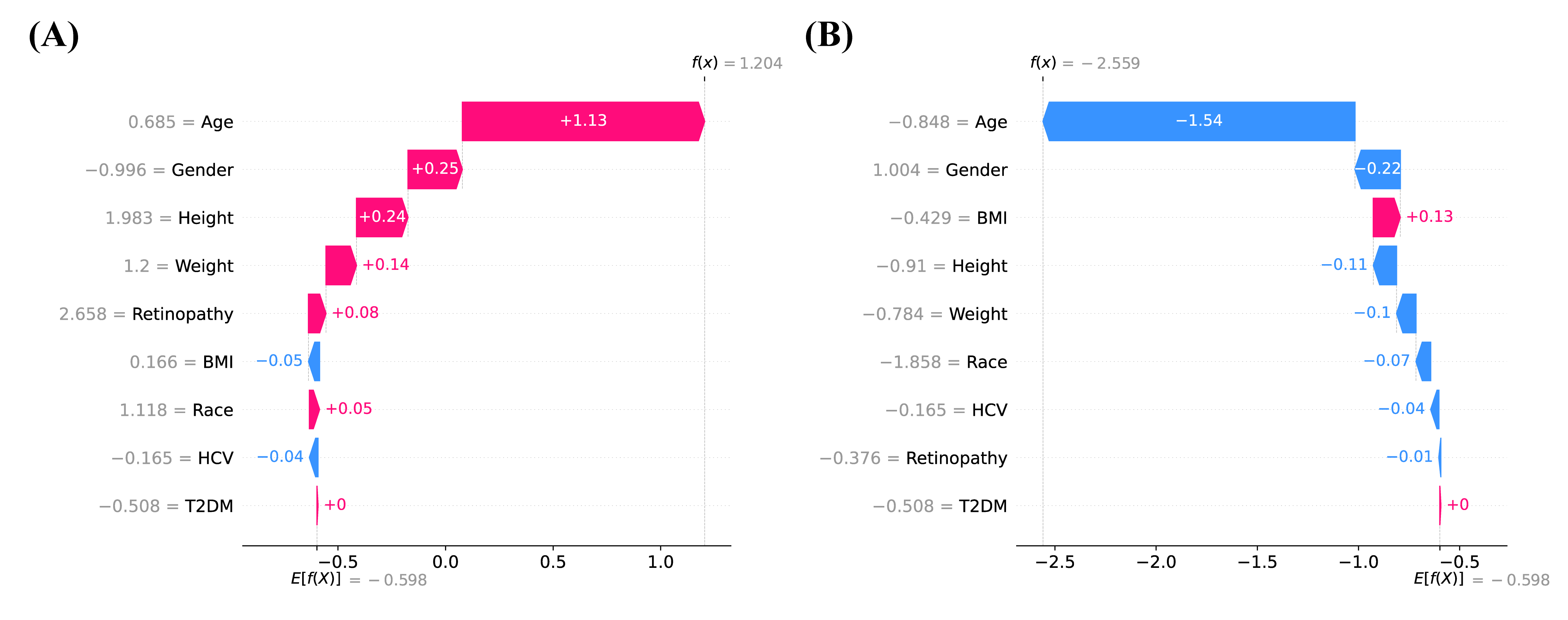

Supplement: Supplementary file 2 — Supplementary Material 2 [file 12911_2024_2749_MOESM2_ESM.docx]
