# Supplementary material for: Explainable machine learning model for predicting the risk of significant liver fibrosis in patients with diabetic retinopathy
Source: BMC Med Inform Decis Mak. 2024 Nov 11;24:332. doi: 10.1186/s12911-024-02749-z (PMC11552118; doi:10.1186/s12911-024-02749-z)
Supplement: Supplementary file 4 — Supplementary Material 4 [file 12911_2024_2749_MOESM4_ESM.docx]

**Table S1. Pairwise Comparison of ROC Curves for Different Classification Models Using DeLong's Test**

| Model | Z-value | p-value |
| --- | --- | --- |
| LR vs. RF | 5.074 | **<0.001** |
| LR vs. NB | 5.493 | **<0.001** |
| LR vs. SVM | 4.211 | **<0.001** |
| LR vs. XGBoost | 5.339 | **<0.001** |
| LR vs. MLP | 3.634 | **<0.001** |
| LR vs. KNN | 9.898 | **<0.001** |
| LR vs. DT | 15.381 | **<0.001** |

Abbreviations: XGBoost, Extreme Gradient Boosting; RF, Random Forest; MLP, Multilayer Perceptron; SVM,

Support Vector Machine; LR, Logistic Regression; NB, Naive Bayes; DT, Decision Tree; KNN, K-Nearest Neighbors;
