# Supplementary material for: Explainable machine learning model for predicting the risk of significant liver fibrosis in patients with diabetic retinopathy
Source: BMC Med Inform Decis Mak. 2024 Nov 11;24:332. doi: 10.1186/s12911-024-02749-z (PMC11552118; doi:10.1186/s12911-024-02749-z)
Supplement: Supplementary file 5 — Supplementary Material 5 [file 12911_2024_2749_MOESM5_ESM.docx]

1. **Comparison of Machine Learning Models and Their Parameter Settings: XGBoost, Decision Tree, Logistic Regression, MLP, Naive Bayes, KNN, Random Forest, and SVM**

**XGBoost:** boost_tree(

trees = 100,

tree_depth = 6,

learn_rate = 0.3,

loss_reduction = 0,

sample_size = 1,

mtry = NULL )

**Decision Tree:** decision_tree(

cost_complexity = 0.01,

tree_depth = NULL,

min_n = 20)

**Logistic Regression:** logistic_reg(

penalty = 0,

mixture = 0)

**MLP:** mlp(

hidden_units = 5,

penalty = 0,

epochs = 100)

**Naive Bayes:** naive_Bayes(

smoothness = 1,

Laplace = 0)

**KNN:** nearest_neighbor(

neighbors = 5,

weight_func = "rectangular",

dist_power = 2)

**Random Forest:** rand_forest(

mtry = sqrt(ncol(train_data)),

trees = 500,

min_n = 1)

**SVM:** svm_rbf(

cost = 1,

rbf_sigma = NULL

)

1. **A Comprehensive Workflow for Data Analysis and Machine Learning Using SPSS, R, and Python: From Data Cleaning to SHAP Interpretability**

In this study, I utilized SPSS software (Version 23.0), R software (Version 3.3.2), and Python software (Version 3.10.4) to conduct a multi-step data analysis and machine learning workflow. The process began by downloading the relevant source files from the NHANES database, followed by data reading, organization, and cleaning in R. Data cleaning is a critical step to ensure consistency and accuracy, providing a robust foundation for subsequent analysis. Once the data was cleaned, I conducted basic statistical analyses in SPSS, such as creating a baseline table (table1) using t-tests and chi-square tests, which are suited for assessing group differences in descriptive statistics and initial feature analysis.

Next, I employed the Boruta package in R for feature selection. This method, based on the Random Forest algorithm, is highly effective in identifying significant features, thus optimizing model performance. Through the Boruta algorithm, I identified nine variables significantly associated with the outcome variable, as illustrated in Figure 2.

After completing feature selection, I proceeded to build machine learning models in R. Initially, I divided the dataset into training and testing sets using stratified sampling to ensure balanced representation of target classes. A random seed was set to maintain reproducibility throughout the analysis. I then standardized the features to ensure comparability across different scales. A total of eight machine learning models were developed, starting with a parameter tuning phase to identify the optimal parameter combinations. Model performance was further enhanced by refining these parameters, employing a 10-fold cross-validation approach for robust resampling. This process identified the best-performing model, which was logistic regression. I visualized its results with additional diagnostic tools, including a confusion matrix (Supplementary Figure 1), as well as ROC curves, DCA curves, calibration curves, and PR curves (Figures 3 and 4). Finally, I calculated the predictive performance of each model on both the training and validation sets (Tables 2 and 3), allowing for a comprehensive assessment of model accuracy and generalizability.

Finally, SHAP (SHapley Additive exPlanations) analysis was implemented in Python, a widely used approach for interpreting machine learning model outputs. This analysis assigned each feature a specific contribution value to the model's predictions, significantly enhancing the interpretability and transparency of our model (Figure 5). Additionally, through Python, predictions for true negative and true positive patients were conducted (Supplementary Figure 2). Finally, an online web-based calculator was developed using R’s Shiny package to facilitate clinical application and ease of use (Supplementary Figure 3).

This workflow is methodologically sound and ensures that each software tool contributes its strengths at different stages of the analysis, guaranteeing both the accuracy of the data analysis and the interpretability of the machine learning model.
